# Within, but not between hands interactions in vibrotactile detection thresholds reflect somatosensory receptive field organization

**DOI:** 10.3389/fpsyg.2014.00174

**Published:** 2014-02-28

**Authors:** Luigi Tamè, Andrew Moles, Nicholas P. Holmes

**Affiliations:** ^1^Centre for Integrative Neuroscience and Neurodynamics, School of Psychology and Clinical Language Sciences, University of ReadingReading, UK; ^2^Center for Mind/Brain Sciences, University of TrentoRovereto, Italy

**Keywords:** tactile, fingers, detection thresholds, QUEST

## Abstract

Detection of a tactile stimulus on one finger is impaired when a concurrent stimulus (masker) is presented on an additional finger of the same or the opposite hand. This phenomenon is known to be finger-specific at the within-hand level. However, whether this specificity is also maintained at the between hand level is not known. In four experiments, we addressed this issue by combining a Bayesian adaptive staircase procedure quick estimation of threshold (QUEST) with a two-interval forced choice (2IFC) design in order to establish threshold for detecting 200 ms, 100 Hz sinusoidal vibrations applied to the index or little fingertip of either hand (targets). We systematically varied the masker finger (index, middle, ring, or little finger of either hand), while controlling the spatial location of the target and masker stimuli. Detection thresholds varied consistently as a function of the masker finger when the latter was on the same hand (Experiments 1 and 2), but not when on different hands (Experiments 3 and 4). Within the hand, detection thresholds increased for masker fingers closest to the target finger (i.e., middle > ring when the target was index). Between the hands, detection thresholds were higher only when the masker was present on any finger as compared to when the target was presented in isolation. The within hand effect of masker finger is consistent with the segregation of different fingers at the early stages of somatosensory processing, from the periphery to the primary somatosensory cortex (SI). We propose that detection is finger-specific and reflects the organization of somatosensory receptive fields in SI within, but not between the hands.

## INTRODUCTION

Detecting a tactile stimulus on our body is one of the most basic tasks accomplished by our brain in the context of tactile perception. However, even though this phenomenon is simple it requires a series of quite elaborate processing that involves the somatosensory as well as other brain systems. From the classical studies conducted by Fritsch and Hitzig in dogs (Fritsch and Hitzig, 1870), and by Penfield and colleagues in humans (Penfield and Boldrey, 1937), we know that basic tactile inputs, at least from one side of the body, are represented somatotopically. More recent neurophysiological studies in animals (Powell and Mountcastle, 1959; Iwamura et al., 1993), as well as behavioral (Schweizer et al., 2001) and neuroimaging studies in humans (Overduin and Servos, 2004; Nelson and Chen, 2008; Martuzzi et al., 2014) have corroborated this. Moreover, evidence suggests that a somatotopic organization of tactile information arising from the fingers may also occur for stimuli coming from both sides of the body simultaneously (Braun et al., 2005). In this respect, neurophysiological studies in animals have demonstrated the presence of bilateral receptive fields in Brodmann’s area 2 of monkeys (Iwamura et al., 2001), part of the homologous primary somatosensory cortex (SI) in humans. Similarly, neuroimaging data from our laboratory recently found finger-specific blood-oxygenation level-dependent (BOLD) responses in SI, when vibrotactile stimuli were delivered on the two sides of the body suggesting the capability of this area of integrating bilateral tactile stimuli (Tamè et al., 2012).

Behaviourally, tactile perception of stimuli on the fingers of the same and different hands has been investigated using tasks involving mislocalization (e.g., Schweizer et al., 2001), memory (e.g., Harris et al., 2001), and masking (Uttal, 1960; Tamè et al., 2011). The masking paradigm relies on the interference generated by the presentation of two tactile stimuli (or patterns of stimulation) on the body simultaneously, or in close temporal proximity. This task has been used successfully to investigate many aspects of tactile processing (Laskin and Spencer, 1979), including the detection (Gilson, 1969) and identification of patterned stimuli (Craig and Xu, 1990), and the effects of location, hand preference (Verrillo et al., 1983), delay (Craig, 1983), duration (Gescheider and Migel, 1995), and intensity (Craig, 1974). In this respect, Sherrick (1964) showed that within the hand, the interference was greater when the masker and the target were on the same (i.e., right index finger) compared to different fingers (right index and little fingers). To a lesser degree, the interference was present also when the masker and the target were on fingers of different hands (Sherrick, 1964). Similarly, Gescheider et al. (1970) reported masking when homologous fingers of the two hands were stimulated together (Gescheider et al., 1970). However, despite this early (Sherrick, 1964; Gescheider et al., 1970) and more recent (Craig, 1985; Craig and Qian, 1997) evidence on the representation of the fingers within and between the hands, it remains unclear whether within and between hands interactions in the detection of vibrotactile stimuli on different fingers reflect the detailed organization of somatosensory receptive fields – i.e., whether masking is greater for fingers that may share a representation up to and including SI.

Here, we investigated whether tactile detection thresholds for stimuli on a pre-specified target finger can be modulated by a simultaneous tactile stimulus applied on another finger, either on the same (i.e., within) or a different (i.e., between) hand*.* Differently from previous reports, we aimed to determine whether simple detection of vibrotactile stimuli at the fingers, in the presence of a masker stimulus, follows a somatotopic receptive field organization both within and between the hands.

## EXPERIMENT 1

First, we investigated tactile detection thresholds on a target finger when the maskers were applied, in different blocks, on another finger of the same hand. On the basis of previous literature (Schweizer et al., 2001), we predicted that the presence of a masker will increase the detection threshold at the target finger. Moreover, this increment should be higher for smaller as compared to larger somatotopic distances between the target and masker fingers, as suggested by a previous report (e.g., Sherrick, 1964).

### MATERIALS AND METHODS

#### Participants

Twenty-eight participants (mean ± SD = 24.5 ± 5.2 years; 19 females) took part in the study. Participants of all experiments reported normal or corrected to normal vision and normal touch. All participants in all experiments gave their informed consent prior to participation. The study was approved by the ethics review board of the University of Reading and was carried out according to the principles of the 1964 Declaration of Helsinki (as updated in Seoul, 2008). In Experiment 1, eight participants were discarded from the analysis because they were not able to perceive the vibrotactile stimulation on all the fingers at any intensity. Two additional participants were discarded because of a too high threshold for the single index finger stimulation (such that, in the presence of maskers, thresholds were at ceiling). All participants included into the study were right-handed by self-report.

#### Apparatus and stimuli

Vibrotactile stimuli were delivered to the middle, ring, little, and/or index fingers of one hand using two stimulators (Oticon, Xiamen Bone conductor BC461-1 polarized). The stimulators were driven by a standard PC audiocard (Vinyl AC’97 Audio wave). Tactile stimulation consisted of a 100 Hz sinusoidal wave, fed into the stimulator(s) for 200 ms, with a linear rise and fall of 5 ms. Tactile stimulators were gently pressing the fingers, resting above the table on two levers to which a series of pulleys, cords, and weights were connected. In this way, the stimulators exerted a similar pressure on all fingers, despite finger movements.

Two light-emitting diodes (LEDs) were placed in front of the participant’s hands and indicated the onset of the stimulation intervals. Two foot-response pedals were positioned under the participant’s feet. Stimulus presentation and response collection were controlled by a custom program written using MATLAB R2006b (Mathworks, Natick) and Psychtoolbox libraries (Brainard, 1997). Throughout the experiment, white noise was presented over closed-ear headphones (Pro-Luxe, PX-921 Stereo Headphones), connected to an amplifier (ROTEL Stereo integrated amplifier RA-921) and a custom-built white noise generator, to mask any sounds made by the tactile stimulators. The apparatus and stimuli were identical in all four experiments.

#### Design

**FIGURE 1:**
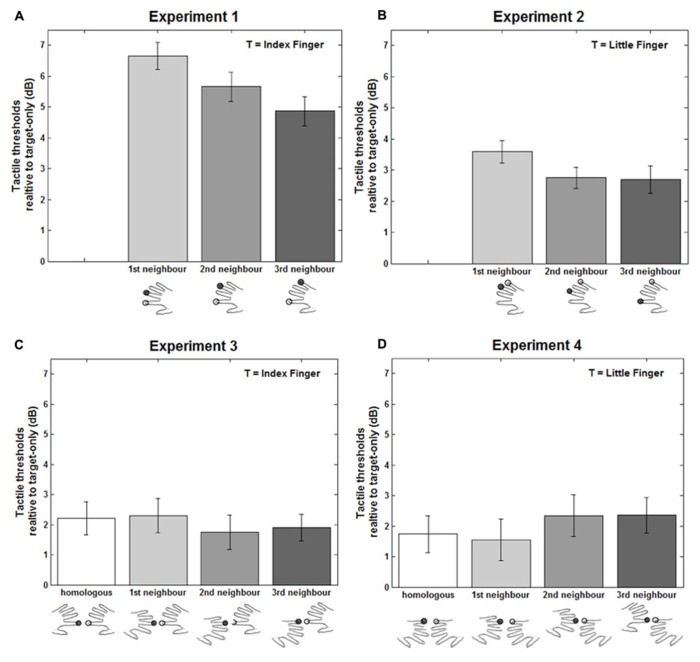
**Results of the four experiments.** Vibrotactile detection thresholds in the double simultaneous stimulation conditions for Experiment 1 **(A)**, 2 **(B)**, 3 **(C)**, and 4 **(D)**, expressed in decibel (dB) relative to the target-only condition. The stimulated target finger (T) is indicated by open circles, while the filled black circles represent the non-target. Error bars represent the standard error of the mean (± SEM) across participants.

The experiment followed a repeated-measures design with four conditions. The labels in **Figure 1A** illustrate all of the possible stimulation conditions for Experiment 1, in which the target finger was the right index and the masker was a finger of the same hand. An empty circle indicates the target finger, whereas a solid filled circle indicates the masker finger. The designated stimulated hand was counterbalanced across participants: half of the participants performed the task with the left index as target finger and the other with the right index finger as target. Each block comprised 63 trials, resulting in a total 252 trials for each participant.

#### Procedure

Before the main experiment, each participant performed about 20 practice trials starting from the maximum possible intensity. The possible intensity at which the fingers were stimulated ranged between 0 and 1, corresponding to the minimum and maximum intensity produced by the computer’s audio card, with voltage output linearly dependent on the signal input. The vibrotactile stimulators were calibrated by recording their output with a microphone. The root mean square output was marginally better-fit as an exponential (*r*^2^ = 0.814) than a linear (*r*^2^ = 0.801) function of the input voltage. These fits are not perfect due to the background noise in the room. Despite this imprecision, we have found our simple setup to be very reliable over several years and will describe these methods in more detail elsewhere (Tamè et al., in preparation). The exponential trend in the stimulators’ input–output function was compensated for by linearising the threshold data (reversing the equation). All statistical analyses were performed both on the raw and the linearised data, and none of the results were affected by this transformation.

The starting intensity for experimental blocks was set for all participants at 0.5, half of the maximum possible intensity. Pilot study revealed that this was the ideal starting point for threshold estimation. Stimulus amplitude was automatically adjusted in accordance with the quick estimation of threshold (QUEST) algorithm on the basis of the participant’s responses (Watson and Pelli, 1983). The default settings of QUEST in the PsychophysicalToolBox3 were used.

Participants were informed that they had to perform a two-interval forced choice (2IFC) task to indicate in which of two temporal intervals the target finger (i.e., index) was stimulated. The target finger was always present in only one interval (i.e., first or second). In the double simultaneous stimulation (DSS) conditions the masker finger was always stimulated in both intervals, with the same waveform as the target and a constant intensity of 0.6. Participants were instructed to keep two foot-pedals pressed, unless indicating the presence of a target in the first (raise the left foot) or second (raise the right foot) interval. Participants could see their hands, and were instructed to keep their gaze between the two LEDs in all experiments to control for gaze position (Harrar and Harris, 2009).

Each trial started with the left LED flashing for 250 ms indicating the potential occurrence, after 500 ms, of the *first* tactile stimulation (1st interval). 500 ms after the end of the first stimulation the right LED flashed (250 ms), indicating the potential occurrence, after 500 ms, of the *second* tactile stimulation (2nd interval). The target finger was always the same and the masker finger was one of the fingers on the same hand, depending on the block. 500 ms after the second interval, both LEDs switched on until the participant gave their response. Participants responded according to whether the target finger was stimulated in the first or second interval as accurately as possible. No feedback about accuracy was provided. Participants were allowed short breaks between blocks. The experimenter remained in the room throughout the session to ensure that participants complied with the instructions.

The detection threshold values were converted to decibels (dBs) relative to the target-only condition using the following formula: dB = 10^*^log_10_(DSS threshold/target-only threshold; for a similar conversion method see D’Amour and Harris, 2013). All of the experimental programs, the raw data, and analysis scripts are or will be available on the laboratory’s webpages (http://neurobiography.info/handlab.php).

## RESULTS

The masker was presented at a M ± SE 6.05 ± 0.328 dB relative to participants’ thresholds in the target only condition. The threshold values for the three masker fingers were entered into a one-way analysis of variance (ANOVA) with FINGER as the within-participant variable. Two-tailed paired *t*-tests were used for all planned comparisons. This analysis revealed a significant main effect of FINGER, *F*(2,34) = 13.07, *p* < 0.0001, MSE = 1.1, η^2 ^= 0.44. As shown in **Figure 1A**, all DSS stimulation conditions were significantly increased relative to the target-only condition [1st: M ± SE = 6.65 ± 0.433 dB, *t*(17) = 15.35, *p* < 0.0001; 2nd: M ± SE = 5.66 ± 0.475 dB, *t*(17) = 11.91, *p* < 0.0001; 3rd: M ± SE = 4.87 ± 0.470 dB, *t*(17) = 10.37, *p* < 0.0001]. When the masker was the middle finger (1st neighbor) participants’ detection threshold was also significantly higher compared to when the ring [2nd neighbor: *t*(17) = 3.01, *p* = 0.008] and the little [3rd neighbor: *t*(17) = 5.01, *p* = 0.0001] fingers were the maskers. Finally, threshold was also higher when the ring (2nd neighbor) as compared to the little finger [3rd neighbor: *t*(17) = 2.18, *p* = 0.044] was the masker.

## DISCUSSION

In Experiment 1, we found that tactile detection threshold was lower (i.e., better performance) when the index finger (i.e., target) was stimulated in isolation compared to when another (i.e., masker) finger was simultaneously stimulated. This is in accordance with previous reports on tactile DSS (Craig, 1985; Evans and Craig, 1991; Tamè et al., 2011, 2013) showing interference in tactile detection with simultaneous maskers. More interestingly, and also as expected, threshold on the index finger was higher when the masker was a closer (i.e., D2 with D3) than a further finger (i.e., D2 with D5); a decreasing threshold with increasing distance between the fingers. This finding likely reflects the partial overlap of the tactile receptive fields of adjacent fingers in early somatosensory processing (Iwamura et al., 1980), and speaks in favor of a somatotopic organization for the detection of vibrotactile stimuli at the fingers with maskers on the same hand. Similar results were found by Schweizer et al. (2001) using a mislocalization task – digits closer to the stimulated one received a higher number of mislocalizations compared to digits further away (Schweizer et al., 2001).

However, there is the possibility that the effect we reported is in part due to a special relation of the index finger (i.e., the target) with respect to the other, masker fingers. This issue will be addressed in experiment 2.

## EXPERIMENT 2

It is possible that the results of Experiment 1 may in part derive from the special role played by the index finger, rather than a more general organizational structure of finger representations. Indeed, some work has shown different perception of the index compared to the other fingers. Vega-Bermudez and Johnson (2001) found significantly reduced spatial acuity from the index to the middle and also from the middle to the ring finger. Neuroimaging studies have shown that human SI has multiple representations of the index finger (Sanchez-Panchuelo et al., 2012). By contrast, other studies have failed to find differences between the index and middle fingers in the context of tactile DSS (Tamè et al., 2011, 2013). Therefore, it remains unclear whether the effect found in Experiment 1 reflects somatotopic organization, or is instead contaminated by the index finger’s peculiarity. In Experiment 2, the little finger was the target finger. If the detection of a stimulus with maskers on fingers of the same hand follows a somatosensory receptive field organization, we should find a greater increase in the detection threshold when the masker is the ring (i.e., 1st neighboring finger) than the middle or index fingers. Instead, if the organization depends on a special role played by the index finger, we should expect a similar profile to Experiment 1, with a higher detection threshold when the masker is the middle finger (in the present experiment the 2nd neighboring finger).

### MATERIALS AND METHODS

#### Participants

Twenty-one participants were recruited but one was discarded from the analysis, because he later reported problems with tactile sensation in his left hand. The final sample was 20 (mean ± SD age = 26.5 ± 5.3 years; 13 females, 17 right-handed). Improvements in our recruitment and testing procedures meant that none of the participants were excluded for being unable to perceive the stimuli.

#### Design and procedure

This was identical to Experiment 1, with the following exceptions. The target finger was the little finger and the maskers were the ring (1st neighbor), middle (2nd neighbor), and index (3rd neighbor) fingers, respectively. To help participants to learn the task and detect the stimuli feedback was provided by both LEDs flashing twice after incorrect responses. This choice was dictated by the fact that, unlike many psychophysical experiments, we mostly recruited relatively naïve and untrained participants. Therefore, this modification was introduced with the intent of avoiding ceiling effects and participant exclusion due to the difficulty of the task for some participants. The experiment comprised two sessions separated by a 5 min break; each block was repeated twice. Blocks comprised 48 trials each, resulting in a total of 384 trials per participant.

## RESULTS

The masker was presented at a M ± SE 4.65 ± 0.410 dB relative to participants’ thresholds in the target only condition. Detection thresholds in the DSS trials in dB relative to the target-only trials are reported in **Figure 1B**. A one-way ANOVA with FINGER as the within-participant variable revealed a main effect of FINGER [*F*(2,38) = 5.32, *p* = 0.009, MSE = 0.94, η^2^ = 0.22]. As shown in **Figure 1B**, detection thresholds in all the DSS conditions, as in Experiment 1, were higher [1st neighbor: *t*(19) = 9.96, *p* < 0.0001; 2nd neighbor: *t*(19) = 7.97, *p* < 0.0001; 3rd neighbor: *t*(19) = 6.25, *p* < 0.0001] compared to the target-only condition. Importantly, the DSS condition in which the ring finger (here the 1st neighbor) was the masker showed a significantly greater detection threshold (M ± SE = 3.59 ± 0.360 dB) compared to when the middle [2nd neighbor: M ± SE = 2.75 ± 0.346 dB, *t*(19) = 2.72, *p* = 0.01] and index [3rd neighbor: M ± SE = 2.70 ± 0.431 dB, *t*(19) = 3.01, *p* = 0.007] were the masker fingers. No other comparisons were significant (All ps > 0.77).

## DISCUSSION

Experiment 2 tested whether the results of Experiment 1 derived, at least in part, from a special role played by the index finger. In accordance with Experiment 1, vibrotactile detection thresholds were higher for all the DSS conditions compared to the target-only detection condition. Moreover, and supporting our hypothesis concerning somatosensory receptive field organization, vibrotactile detection thresholds were affected differently as a function of the masker finger. Thresholds were higher when a closer (i.e., 1st neighbor, ring finger) compared to a further (i.e., 2nd and 3rd neighbors; middle and index fingers) masker finger with respect to the target was stimulated. This result rules out the possibility that the effect we reported in Experiment 1 was derived from a special role played by the index finger, and supports a somatotopic receptive field organization for the detection of vibrotactile stimuli at the fingers, with maskers delivered within the same hand. However, differently from Experiment 1, the amount of interference was the same regardless of whether index or middle fingers were the maskers, showing a relatively greater interference effect produced by the index finger as one might expect based on the physical distance between the target and the masker (i.e., little and index fingers). This result may somehow reflect a particular sensitivity in acting as a masker for the index finger.

## EXPERIMENT 3

Having assessed the organization of the fingers’ interactions within the same hand (Experiments 1 and 2), Experiment 3 aimed to examine the interaction between the fingers of the two hands. In this experiment, the masker finger was always on the hand opposite the target finger. If the fingers’ interaction reflects the somatosensory receptive field organization also between the hands, we should have the same pattern of results as Experiment 1, with a different level of interference depending of the somatotopic distance between the target and masker fingers. Instead, if such organization is not maintained across the hands, we should not see any difference in the level of interference as a function of the somatotopic distance between the masker and target fingers stimulated.

### MATERIALS AND METHODS

#### Participants

15 participants (mean ± SD age = 27 ± 4 years; 10 females) took part in the study. Thirteen were right-handed by self-report, two were left-handed. None were excluded for poor performance.

#### Design and procedure

This was identical to Experiments 1 and 2, with the following exceptions. The target finger was always the index finger, whereas the maskers were the fingers of the opposite hand with respect to the target. In particular, depending on the stimulation condition, the masker was the index (i.e., the finger homologous to the target), the middle, the ring, or the little fingers. The first two participants did two sessions of 48 trials per condition, whereas the rest did 36 trials per condition per session, to reduce the overall length of the experiment.

## RESULTS

The masker was presented at a M ± SE 8.69 ± 0.453 dB relative to participants’ thresholds in the target only condition. Detection thresholds in the DSS trials, as for Experiments 1 and 2, were expressed relative to the target-only condition, pooled across left and right target hands, and entered into a one-way ANOVA with FINGER as the within-participant variable. The analysis revealed no significant main effect of FINGER [*F*(3,42) = 0.55, *p* = 0.65, MSE = 1.79, η^2^ = 0.04]. Differently from Experiments 1 and 2, between hands stimulation did not show any difference in the vibrotactile detection thresholds as a function of the stimulated fingers of the opposite hand with respect to the target. However, as shown in **Figure 1C**, all the DSS stimulation conditions were significantly different from the target-only condition [1st: M ± SE = 2.22 ± 0.549 dB, *t*(14) = 4.04, *p* = 0.001; 2nd: M ± SE = 2.29 ± 0.521 dB, *t*(14) = 4.39, *p* = 0.001; 3rd: M ± SE = 1.75 ± 0.571 dB, *t*(14) = 3.07, *p* = .008; 4th: M ± SE = 1.90 ± 0.435 dB, *t*(14) = 4.36, *p* = 0.001]. Therefore, as in the within hand stimulation where the target and masker fingers were on the same hand, also in this experiment when the masker was on the other hand this caused a general interference effect, even though it was not affected by masker finger.

## DISCUSSION

Experiment 3 examined the possibility of a somatotopic receptive field organization for the detection of vibrotactile stimuli delivered on both hands. If the somatotopic organization is preserved we should still see a modulatory effect in the detection threshold of the target as a function of the masker finger stimulated on the opposite hand.

In accordance with Experiments 1 and 2, we found an increase in the vibrotactile detection thresholds when the masker was present compared to when the target finger was stimulated in isolation. However, different from the previous experiments, in which the target and masker fingers were on the same hand, we did not find any effects on the detection threshold of the target finger as a function of the masker finger. Therefore, these data speak in favor of a non-somatotopic receptive field organization for detecting vibrotactile stimuli with maskers on the opposite hand.

## EXPERIMENT 4

Having assessed the effects on tactile thresholds when stimuli were delivered on the two hands with the index finger as target, as in the within hand stimulation conditions (Experiments 1 and 2), for completeness we tested for any possible modulatory effects when a different finger was the target. Therefore, the little finger was adopted as a target in place of the index finger, whereas the maskers remained the four fingers of the other hand as in Experiment 3. Paired comparisons were two-tailed, due to the lack of a strong prediction, and the absence of significant effects in Experiment 3.

### MATERIALS AND METHODS

#### Participants

15 participants (mean ± SD age = 26 ± 5 years; 10 females) took part in the study. Thirteen were right-handed by self-report, two were left-handed. None were excluded for poor performance.

#### Procedure

This was identical to Experiment 3, with the following exceptions. The target finger was always the little finger, whereas the maskers were the fingers of the opposite hand with respect to the target. In particular, depending on the stimulation condition, the masker was the little (i.e., homologous to the target), the ring (1st neighboring), the middle (2nd neighboring), or the index (3rd neighboring) fingers. As in Experiment 3, participants performed 36 trials per condition for each of two sessions separated by a 5 min break.

## RESULTS

The masker was presented at a M ± SE 8.66 ± 0.509 dB relative to participants’ thresholds in the target only condition. The detection threshold values for the DSS condition in dB relative to the target only condition were entered into a one-way ANOVA with FINGER as within-participant variable. This analysis revealed no significant effect of FINGER [*F*(3,42) = 1.99, *p* = 0.129, MSE = 1.30, η^2^ = 0.13]. As for Experiment 3, we did not see any differences in the detection threshold as a function of the masker finger. However, as shown in **Figure 1D**, all the DSS stimulation conditions were significantly different from the target-only condition [1st: M ± SE = 1.74 ± 0.600 dB, *t*(14) = 2.91, *p* = 0.012; 2nd: M ± SE = 1.55 ± 0.512 dB, *t*(14) = 3.02, *p* = 0.009; 3rd: M ± SE = 2.35 ± 0.685 dB, *t*(14) = 3.42, *p* = 0.004; 4th: M ± SE = 2.36 ± 0.583 dB, *t*(14) = 4.04, *p* = 0.001]. This experiment replicates the pattern of results of Experiment 3, showing a decrement in the performance (i.e., higher detection thresholds) for all the DSS conditions compared to single finger stimulation and the same pattern between the DSS conditions.

## DISCUSSION

Experiment 4 tested for any modulatory effects on vibrotactile detection thresholds when the target was on the little finger and the maskers were on fingers of the opposite hand. In accordance with Experiment 3, we found an increase in threshold for all the stimulation conditions in which the masker was present compared to that in which the target finger was stimulated in isolation. Moreover, in accordance with Experiment 3, we did not find any modulatory effects on the threshold of the target finger as a function of the masker finger (i.e., 1st, 2nd, 3rd, or 4th neighboring finger). This supports the notion of a non-somatotopic receptive field organization for the detection of vibrotactile stimuli with maskers on the opposite hand.

## GENERAL DISCUSSION

In the present work we examined whether detection of vibrotactile stimuli at the fingers in the presence of maskers follows a somatotopic receptive field organization both within and between the hands. In Experiment 1, we tested the detection threshold of a target finger (i.e., index finger), while in different blocks a masker finger of the same hand was simultaneously stimulated. In Experiment 2, we did the same with the exception that the target was the little in place of the index finger. In Experiments 3 and 4, the masker was always a finger of the opposite hand with respect to the target, which was the index and little finger, respectively.

### WITHIN HAND FINGER REPRESENTATION FOR VIBROTACTILE DETECTION THRESHOLDS

Experiments 1 and 2 showed that vibrotactile detection thresholds for a pre-specified target finger increase when a concurrent masker is presented on another finger of the same hand. These results are in accordance with previous reports on tactile DSS at the fingers (e.g., Evans and Craig, 1991; Tamè et al., 2011) that showed impairment in participants’ performance when two tactile stimuli are presented simultaneously compared to when a single stimulus was presented. This reduction in the participants’ performance derives from the competition generated by the two tactile stimuli to be represented (Verrillo et al., 1983; Johansen-Berg and Lloyd, 2000). Most importantly, the detection threshold of the target finger varied as a function of the masker finger. The threshold was greater the smaller the distance between the target and masker fingers (see **Figure 1A**). In particular, when the target was the index finger (i.e., Experiment 1), maskers on the middle caused a greater increment in the detection threshold compared to when the masker was the ring, which in turn was greater than when the masker was the little finger. This result supports the notion of a somatotopic organization of finger representation within the hand for simple tactile detection. This is an expected result in agreement with the classical human (Penfield and Boldrey, 1937) and more recent monkey (Merzenich et al., 1987; Iwamura et al., 1993) neurophysiological studies. This is also compatible with Sherrick’s (1964) report in which tactile DSS produced greater masking when the same finger was stimulated twice (i.e., index) compared to when target and masker were on different fingers (i.e., index and little fingers). Similarly, other behavioral studies in humans found that both the strength of tactile interference between the fingers of the same hand (Schweizer et al., 2001) and perceptual learning (Sathian and Zangaladze, 1997; Harris et al., 2001; Harrar et al., 2014) depend on the proximity of the stimulated fingers. For instance, Schweizer et al. (2001), using a mislocalization task in which participants had to localize tactile stimuli on the fingers, found that the errors were somatotopically distributed. In addition to behavioral (e.g., Uttal, 1960; Haggard et al., 2006), neuroimaging (Nelson and Chen, 2008; Sanchez-Panchuelo et al., 2010; Braun et al., 2011) studies in humans revealed a somatotopic representation of tactile stimuli on the fingers of the same hand. Martuzzi et al. (2014), using a 7T functional magnetic resonance imaging (fMRI) experiment, showed that the representation of the fingers in the SI follows a somatotopic organization (Martuzzi et al., 2014; see also Sanchez-Panchuelo et al., 2012).

However, it may be objected that this result is, in part due to a special role played by the index finger with respect to the other fingers. For instance, a recent fMRI study by Schweisfurth et al. (2011), exploring the inter-finger somatotopy in Brodmann’s area (BA) 3b of SI, found that, across participants, the representation of the little, but not the index finger consistently followed a somatotopic organization (Schweisfurth et al., 2011; on the peculiarity of index finger see also Vega-Bermudez and Johnson, 2001). Reasoning that the index may be special led to our Experiment 2, in which the target finger was the little in place of the index finger. The results showed the same pattern revealed in Experiment 1, where maskers on the ring finger (1st neighbor) increased the vibrotactile detection threshold more than masker on the middle finger (2nd neighbor; see **Figure 1B**). However, differently from Experiment 1, the middle and index fingers maskers produced a similar amount of interference. In particular, the reduction of the interference with increasing physical distance (i.e., stimulated fingers) between the target and the masker was less pronounced for the index compared to the middle finger. The combined results of these two experiments suggest that detection of vibrotactile stimuli with maskers within the same hand follows a somatotopic receptive field organization. Moreover, as shown by Experiment 2, this type of organization was not specific to the index finger (Tamè et al., 2011, 2013) as might be expected (Vega-Bermudez and Johnson, 2001; Schweisfurth et al., 2011), but represents instead a general organization of tactile detection on the fingers within the same hand. However, despite the non-finger specificity of the effect, we recognize a particular sensitivity of the index finger when acting as a masker.

Overall, we have shown that, in line with previous literature on this topic, vibrotactile detection with maskers on fingers within the same hand follows a specific somatotopic receptive field organization.

### BETWEEN HANDS FINGER REPRESENTATION FOR VIBROTACTILE DETECTION THRESHOLDS

As in the within, also for the between hands stimulation conditions, we found a large increment in the vibrotactile detection threshold when a masker was presented on the opposite hand with respect to the target finger compared to when the target was presented alone. This interference, however, (M ± SE = 2.02 ± 0.339 dB), was lower compared to when masker and target were fingers of the same hand (M ± SE = 4.30 ± 0.343 dB). This is in accordance with previous reports showing that tactile interference is more pronounced with stimuli on the same compared to different hands (e.g., Evans and Craig, 1991). Moreover, the interference effect, as for the within hand experiments, occurred regardless of whether the target was the index or the little finger (see **Figures 1C,D**), confirming also for the between hands interactions the absence of any finger-specific peculiarity. These results confirm the presence of important relations in the detection of vibrotactile stimuli also on locations of the skin that are not directly adjacent, such as the fingers of the two hands. This cross-body side communication is in accordance with early (Sherrick, 1964; Gilson, 1969; Gescheider et al., 1970) and more recent (Harris et al., 2001; Braun et al., 2005; Tamè et al., 2011; D’Amour and Harris, 2013) reports that showed substantial interactions with stimuli delivered on physically distant body sites. Therefore, our results further support the notion of a bilateral representation of tactile stimuli on the body (Sathian and Zangaladze, 1998; Iwamura et al., 2001; Tamè et al., 2012). Moreover, a non-body-part specific origin of this interference, deriving for instance from an attentional effect is also possible (Holmes et al., 2006). Indeed, we know that attentional factors can affect functional organization of the primary SI by modulating the relation between the fingers (Braun et al., 2002). However, it has been shown that these modulatory effects, when present, do not distort the homuncular organization (Braun et al., 2002). In the absence of significant differences between fingers in Experiments 3 and 4, we cannot firmly conclude that there is no relevant somatotopic organization for this task, nor can we firmly rule out the possibility that general attentional factors were responsible for the relatively small 2 dB masking that we found. We are currently following-up on these findings, and will report further results elsewhere.

However, differently from the within hand stimulation conditions (i.e., Experiments 1 and 2), in Experiments 3 and 4 the detection threshold did not vary as a function of the masker finger (see **Figures 1C,D**) – not showing a somatotopy between the fingers of the two sides of the body. In this respect, previous reports on between hands interactions are quite controversial (Craig, 1985; Evans and Craig, 1991). Indeed, some early studies on tactile DSS have shown masking effects are comparable when homologous and non-homologous fingers of the contralateral hand were stimulated (Sherrick, 1964; Gescheider et al., 1970). Instead, other studies on Braille readers (e.g., Lappin and Foulke, 1973) and others on healthy people (e.g., Gescheider and Verrillo, 1982) found facilitatory effects of bilateral tactile stimulation to the hands. Some others found interference effects, for instance, when participants have to localize a tactile stimulus on a finger of one hand, they make more errors if a concurrent tactile stimulation is presented on the opposite hand (Braun et al., 2005). Moreover, this interference effect appears to be finger-specific (Braun et al., 2005; see also Harris et al., 2001). Similarly, in recent work from our laboratory in which participants had to perform a go/no-go task to detect a supra-threshold tactile stimulus on a pre-specified finger (i.e., index or middle finger), while a masker could occur simultaneously equally probable on another finger of the same or different hand (i.e., middle finger of the same hand or index and middle finger of the opposite hand), we found that, regardless of the hand, there was an interference effect only when the masker finger was the non-homologous finger with respect to the target (Tamè et al., 2011). Instead, in the present work, as we might expect (Harris et al., 2001; Braun et al., 2005; Tamè et al., 2011, 2013), the interference was not modulated by the homology of the finger.

This apparent discrepancy with the more recent investigations may be attributable to the differences in the approach adopted in the previous reports compared to the present study. Indeed, all previous work used different tasks, such as a go/no-go or mislocalization tasks in a context of tactile detection (Braun et al., 2005; Tamè et al., 2011, 2013). For instance, differently from the present study, in Tamè et al. (2011, 2012) work the occurrence of the masker on a certain finger was unpredictable and the two hands were always aligned in a fixed position in space. Furthermore, other studies that found finger-specificity between the hands when investigating tactile perceptual learning have used a tactile discrimination task (e.g., Sathian and Zangaladze, 1997, 1998; Harris et al., 2001; Harrar et al., 2014). In all these previous approaches the tasks used, because of the specific objectives of the investigations, implied the involvement of more complex tactile processing compared to the ones needed to solve our simple vibrotactile detection task (Romo et al., 2012). Therefore, the findings of the present work suggest that simple vibrotactile detection threshold between fingers of the two hands, free of any temporal or spatial modulations, does not reflect a somatotopic receptive field organization.

## CONCLUSION

The results of the present work show that detection threshold for vibrotactile stimuli on the fingers increases when a concurrent masker finger is simultaneously stimulated compared to when a single stimulus is presented. These decrements in the performance are present both when the two stimuli are on the same hand (i.e., within) or on different (i.e., between) hands. Moreover, within the hand, detection thresholds increased more for masker fingers closer to the target finger (i.e., middle > ring when the target was index and ring > middle when the target was little). This is consistent with a finger-specific representation that reflects the organization of somatosensory receptive fields. Instead, between the hands, simple detection threshold increased regardless of the masker fingers stimulated. We propose that simple detection is finger-specific and reflects the organization of somatosensory receptive fields up to and perhaps including SI within, but not between the hands.

## Conflict of Interest Statement

The authors declare that the research was conducted in the absence of any commercial or financial relationships that could be construed as a potential conflict of interest.
